# Forward Vaccinology: CTL Targeting Based upon Physical Detection of HLA-Bound Peptides

**DOI:** 10.3389/fimmu.2014.00418

**Published:** 2014-09-04

**Authors:** Ellis L. Reinherz, Derin B. Keskin, Bruce Reinhold

**Affiliations:** ^1^Laboratory of Immunobiology, Department of Medical Oncology, Dana-Farber Cancer Institute, Boston, MA, USA; ^2^Department of Medicine, Harvard Medical School, Boston, MA, USA

**Keywords:** T cell epitopes, mass spectrometry, vaccines, proteomics, CTL, resident-memory T cells, TCR

## Abstract

Vaccine-elicited cytotoxic T lymphocytes (CTL) recognizing conserved fragments of a pathogen’s proteome could greatly impact infectious diseases and cancers. Enabling this potential are recent advances in mass spectrometry that identify specific target peptides among the myriad HLA-bound peptides on altered cells. Ultrasensitivity of these physical detection methods allows for the direct assessment of peptide presentation on small numbers of tissue-derived cells. In addition, concurrent advances in immunobiology suggest ways to induce CTLs with requisite functional avidity and tissue deployment. Elicitation of high-avidity resident-memory T cells through vaccination may shift the vaccinology paradigm both for preventive and therapeutic approaches to human disease control.

## Vaccines and Infectious Challenges

On a global scale, vaccines have had the greatest impact on human morbidity and mortality from infectious diseases relative to other medical interventions (1). Current FDA-approved vaccines are primarily, if not exclusively, antibody-based in their action, eliciting effective neutralizing antibody responses in pathogen-naïve individuals. Alum-based vaccines are of major clinical utility in facilitating this humoral response. However, these licensed vaccines are protective against pathogens with low antigenic variability. Thus, little to no antigen variation is detected in diphtheria, tetanus, *H. influenzae* B, polio virus, hepatitis A virus, hepatitis B virus, measles, mumps, or rubella viruses. Influenza A virus sequence variability, in contrast, being annual in timescale, requires educated guessing as to appropriate subtype for vaccine formulation to prophylax against future outbreaks. This forecasting approach is only partially successful.

Conventional vaccines against viruses with high mutational rates like hepatitis C virus (HCV) and human immunodeficiency virus-1 (HIV-1), which evolve changes daily under immune selection pressure, or even influenza viruses, which evolve mutations more slowly, will fall short due to intrinsic antibody-evading mechanisms (2). These viruses can avoid attack by immune memory cells through mutation, leaving “yesterday’s” adaptive immune system unable to cope with evolving changes in the viral proteins. Immune escape follows. While it has long been recognized that conserved segments of viral pathogens that cannot mutate due to detrimental effects on viral fitness would be ideal targets against which to engender cross-protective immunity via T cells, the way to implement such an approach has been unclear.

## CTL Targeting

**Figure 1:**
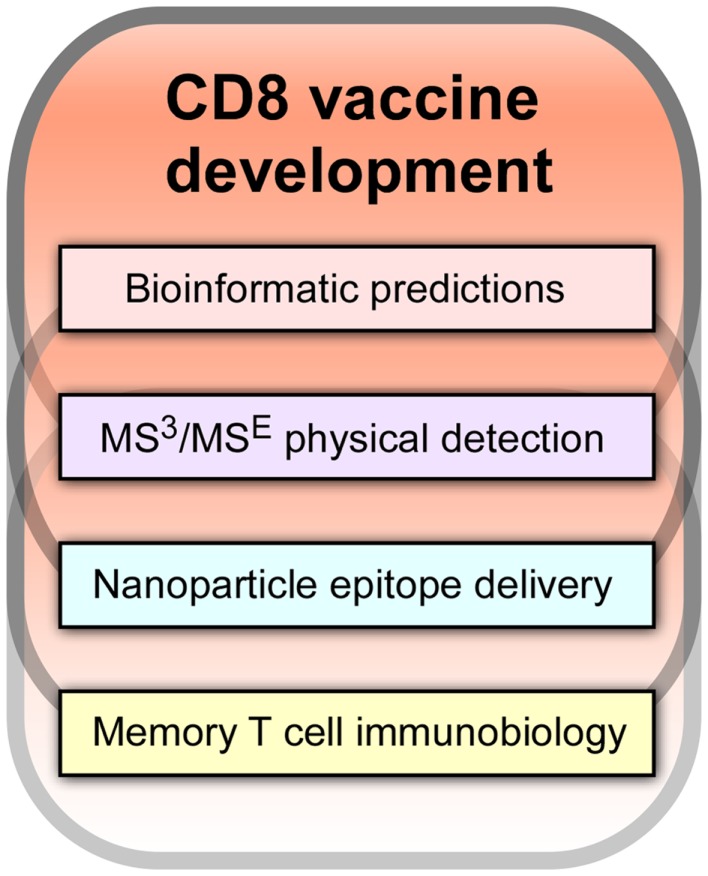
**Essential components of efficacious CD8 vaccine development**.

It is our view that a key path forward involves development of CD8 cytotoxic T lymphocytes (CTL) vaccines. As shown in Figure 1, there are four components of an effective CD8-based T cell vaccine pipeline: (1) facile bioinformatic prediction of conserved segments that include potential T cell epitopes from variable viral strains; (2) physical detection methodologies, most notably mass spectrometry (MS), to determine which of the predicted epitopes are actually arrayed on infected and antigen presenting cells (APCs) for T cell recognition by T cell receptors (TCRs); (3) nanovaccine technology to deliver conserved T cell epitope payloads including adjuvants to APCs for stimulating epitope display in appropriate lymphoid tissue and with optimal display density and time course; and (4) insightful memory T cell biology arising from transcriptomics, proteomics, and other molecular analyses of CD8 subsets defining their development into effector and memory components and the rules guiding their physical deployment into epithelial and lymphoid compartments. Collectively, these technologies and knowledge will create vaccines that elicit potent CD8 memory T cells with effector function that reside at sites of potential viral attack. Such resident-memory T cells (T_RM_) are positioned for immediate action and are in turn aided by subsequent recruitment of T effector (T_EM_) and T central memory cells (T_CM_) from blood and secondary lymphoid tissues. In this way, a prompt immune response is engendered that minimizes viral replication with its pathological consequences. Until very recently, the existence of this important T_RM_ subset with its unique biology was unknown. T_RM_ exhibit a CD103 cell adhesion profile, TGFβ responsiveness and in contrast to circulating CD8 T memory cells, do not need IL-7 and IL-15 for tissue-resident homeostasis (3, 4).

This perspective focuses primarily on physical detection since advances in this area augment CD8 vaccine development efforts in ways that have not been reviewed and are paradigm-shifting. Our view is that the MS approach herein has application to a multiplicity of infectious diseases as well as cancers caused by viruses. Upwards of 20% of tumors worldwide are caused by viruses, with high-risk human papillomaviruses (HPV) alone responsible for >5% of all cancers (5). Computational methods are already available for prediction of peptide binding to multiple HLA alleles, population coverage based on HLA frequencies can be estimated, and there are bioinformatic tools and databases to focus on peptide epitopes conserved among diverse strains of a given virus [(6, 7) and references therein]. Vaccine technology using synthetic materials to target organs, tissues, or cells and deliver concurrently epitopes and immunomodulatory payloads also exist (8).

## Physical Detection

The interaction between a TCR on a CTL and a peptide-bound HLA ligand on an APC [referred to more generally as a peptide-major histocompatibility class I complex (pMHCI) in humans and other mammals] triggers a spectrum of T cell responses in structurally defined terms (9). The molecular definition of the relevant pMHC being recognized is absolutely essential in understanding adaptive immunity. In clinical settings, identification of surface pMHC is generally undetermined or inferred. Although clever use of transgenic clones and engineered infections (10) can circumvent identification of unknown surface pMHC and these methods have led to deep mechanistic insights to rationally develop T cell vaccines, one cannot avoid molecular identification. A primary tool for peptide identification has been to isolate antigen-experienced T cells from some tissue compartment and demonstrate that these cells functionally “recognize” specific pMHCI. Unfortunately, there are many technical issues implementing this reverse immunology approach. For example, *ex vivo* T cell lines that functionally respond to APCs loaded with peptides at high levels may be unable to recognize the low levels of antigen display characteristic of infection or viral transformation. As APC loading levels are defined only by the solution concentration of the peptide with neither the pMHCI density on the loaded APC or on the infected/transformed cell being known, the disparity in T cell avidity between recognizing loaded APCs and recognizing infected cells remains unknown. Beyond technical issues is a fundamental problem: in principle, reverse immunology identifies only antigenic peptides and not those non-antigenic foreign peptides that are displayed as surface pMHCI (*vide infra*).

As MS is the premiere analytical method for the sensitive identification of peptides in complex mixtures, the very limited literature reports of identifying pathogen-derived MHCI/peptide by MS proteomics is notable, but not surprising. The analytical challenge for MS is twofold. First, MHCI peptides are derivatives of total protein metabolism and this complex set is bound to a small set of different MHC proteins on the cell surface. T cells can recognize a few specific pMHCI copies per cell among 100,000 irrelevant other peptides on the same cell (11). Fundamental and practical constraints limit the number of infected or excised cells available for analysis, requiring both high dynamic range and high absolute sensitivity for immunologically useful MS analysis. Second, MHCI peptides are similar in amino acid residue number and hydrophobicity due to MHC-binding requirements resulting in crowding along both the *m*/*z* (mass/charge) axis and the chromatographic elution time axis that characterize peptides and their fragments. For samples like this, standard proteomics can identify thousands of the major components without identifying the immunologically important but quantitatively minor peptides within the pool.

Our laboratory has applied the theory of probabilistic measures for stochastic processes to recognize an ion’s fragmentation pattern when it is set among a complex set of other ion fragments. In general, ion arrival rates in different *m*/*z* channels of the mass spectrometer is a Poisson process and a Poisson measure is used to assign a magnitude of a target molecule’s fragmentation spectrum when it is buried among ion fragments from unrelated molecules. In one format, denoted MS^3^ Poisson detection, the measure has been applied to detecting MHCI peptides without LC separations. Using static nanospray, MS^3^ spectra are generated directly from the complex mixture by first isolating and fragmenting a precursor *m*/*z* and then isolating and fragmenting a selected fragment of the precursor. The MS^3^ spectrum is the mass spectrum of the fragment of a fragment of a molecular ion. Prior to analyzing the mixture, the pure molecule (e.g., synthetic peptide) will have been studied to determine the optimal MS^3^ spectra for detection and these spectra acquired for later use. MS^3^ spectra of the mixture are then acquired with the same *m*/*z* selection steps and the probabilistic measure determines if the target molecule’s MS^3^ spectra are present against the background of other molecules that had the same molecular *m*/*z* and generated the same fragment *m*/*z*. The two *m*/*z* selection steps act somewhat like chromatography in that they simplify the overall ion population while increasing the fraction of the ions that are derived from the target molecule. Two important features have been shown. First, the dynamic range of detection, defined as the target’s fraction of the total ion flux, can be directly estimated and is on the order of 10^5^. Second, the sensitivity of MS^3^ Poisson detection compares well with the most sensitive T cell clone that we have generated. The theory of the method and its use in identifying HPV antigens has been published (12–14).

In a second format, the Poisson measure is used to recognize a target ion’s MS^2^ fragment spectrum against a complex background of other ion fragments in a data-independent acquisition (DIA) format. Here different *m*/*z* ranges of precursor ions are selected and dissociated with the set of *m*/*z* ranges selected covering all precursor ions of interest. When selecting an *m*/*z* interval for fragmentation, ions outside the interval are lost. Better sensitivity is associated with selecting fewer, and therefore, wider, *m*/*z* intervals. However, wider *m*/*z* intervals increase the fragment background relative to the target’s fragments and here the Poisson measure is applied. The Poisson measure of any target is then calculated for each scan and plotted as a chromatogram. In a DIA format, prior knowledge of the target’s MS^2^ spectrum is not required for data acquisition outside of the target ion’s *m*/*z* being contained in at least one of the selected *m*/*z* intervals. Knowledge of the MS^2^ spectrum is required only later when the amassed data are being mined for a specific molecular ion. Using organic monolithic columns with flow rates of 10 nl/min and collecting a series of overlapped *m*/*z* intervals, highly sensitive Poisson detection chromatograms for targets defined post-acquisition can be generated (Poisson segmented LC-MS^E^). Rapid electronic data capture, high resolution mass spectrometers and information-rich precursor, and ion fragment beams allow deep targeted interrogation and re-interrogation of precious samples after the sample is acquired and data archived. Poisson MS^3^ and segmented LC-MS^E^ have very different strengths and are highly orthogonal in the features that they employ for detection. Their combination we have found is considered to be most effective.

## Direct Presentation Versus Cross-Presentation

CD8 T_CM_ and T_EM_ are thought to circulate through blood, interconverting while passing through lymphoid and non-lymphoid tissues (15, 16). Emerging evidence indicates that T_RM_ reside long-term in the brain and mucosal tissues (such as the lungs, gut, and skin) and show only limited levels of egress and recirculation (17). These cells have a characteristic CD103^hi^CD69^hi^CD27^low^ phenotype and may express high levels of granzyme B. The signaling pathways and transcriptome components regulating formation of these heterogeneous populations are coming to light (18). T_RM_ populations have been identified as embedded within barrier epithelial surfaces both in humans and rodents. CD8 T_RM_ cells rapidly acquire cytotoxic function after encountering pMHCI. For viral infection, the outcome at barrier surfaces is a competition between the production of new viral particles by infected host cells vs. immune protection afforded by CTL recognition and destruction of infected or transformed cells. The finite number of T_RM_ cells at mucosal surfaces with limited scanning mobility places a great premium on matching T_RM_ specificities to MHCI display of pathogens on infected cells. T_RM_ cells link antigen specificity with tissue localization, in part through expressing integrins imprinted by draining lymph nodes (LN). Enriching the T_RM_ population specific for pathogens that are likely to be encountered at a given site based on previous encounters makes immunological sense. However, T cell homing only connects the antigen display of professional APCs in draining LN with the antigen *source* in the periphery; it does not identify or instruct which pMHC should be recognized. The display of pathogen determinants in LN is dependent on antigen trafficking and cross-presentation, while the display at barrier surfaces reflects the direct presentation of infection. It is not sufficient that some of the determinants on professional APCs are displayed in common with infected tissue parenchyma. Stimulation of CTL by APC display that is functionally irrelevant for destruction of infected epithelium may even yield positive functional readouts in ELIspot or other assays, but this response is no biomarker of protection.

The objective for CTL vaccination is to establish an optimal, functionally avid T_RM_ population focusing on the MHCI presentation of pathogen determinants displayed at the epithelial barrier of an infected organ. Cytotoxic response should target determinants that are displayed by infected host cells at early time points in order to eliminate these cells prior to significant viral replication. The cellular scale and sensitivity of the aforementioned MS Poisson detection methods permit such identification.

Of note, in cases where HPV-16 infection has induced epithelial transformation and cervical cancer in HLA-A*0201 hosts, only a single epitope from the E7 oncogene product, E7_11–19_, is naturally processed and presented by this allele on those tumor cells. Pointedly, although E7_11–20_ is capable of binding equivalently to this same HLA allele, the 10-mer peptide, unlike the 9-mer, is not displayed on the primary epithelial cells. In contrast, when a large fragment of E7 as a synthetic peptide is exogenously added to HLA-A*0201 professional APCs, the E7_11–20_ is displayed to a very large extent (14). Since T cells are not strongly crossreactive and are particularly specified to a single peptide length (19, 20), this discordance misguides the immune response. It also explains, in significant part, why the 10-mer vaccine was without clinical effect in a therapeutic HPV-16 cancer trial (21).

## CD8 T Cells: Functional Avidity, Elicitation, and Deployment

Functional avidity refers to the sensitivity of a particular T cell to be triggered by pMHC on an APC. High-avidity T cells are capable of recognizing a very small number of pMHC/target cell (i.e., single digit), whereas low-avidity T cells may require hundreds (22–24). Avidity is dependent on the TCR–pMHC interaction, CD8 co-receptor expression, intracellular signaling molecules, and other factors (25). With respect to the TCR, the Vβ and Vα gene repertoire and the nature of the antigen itself both contribute. Unfeatured pMHCI surfaces, like influenza A M1_58–66_ bound to HLA-A*0201, strongly elicit T cells which, although plentiful, comprise a low-avidity immunodominant response (26, 27). As such, display requirements on infected epithelium for activation of those CTL may be at too high a copy number to be achieved during natural infection. Consequently, this CTL response would be non-protective. By using next generation sequencing technologies to identify T cell antigen-specific repertoires and rapid αβ TCR cloning and expression systems (28), their functional TCR-pMHC binding characteristics can be cataloged. In conjunction with quantitation of a given viral pMHC copy number on epithelium or other cells during early infection, the best epitopes can be selected for CTL elicitation through vaccination. In turn, slow release of these epitopes from nanovaccines or other biomaterials formulation (8) in conjunction with routes of administration and chemokines to foster dissemination in relevant tissues (29) can afford optimal protection.

Imagine a time in which CTL-eliciting vaccines are predicated on knowledge of the actual HLA-linked viral peptidome with epitope formulations reflecting peptides conserved in sequence among viral strains and displayed at the earliest time points of infection in numbers sufficient to elicit responding TCRs from the human repertoire. If this could be applied to influenza A, for example, a single vaccine incorporating a few such epitopes would be sufficient, in principle, to afford protection against both seasonal and pandemic variants and bird or swine flu (6). Assuming that we can amalgamate advances in ion physics, structural biology, immunology, computer science, and virology, there is no reason to expect such advances will not be achievable.

## Conflict of Interest Statement

The authors declare that the research was conducted in the absence of any commercial or financial relationships that could be construed as a potential conflict of interest.

## Abbreviations

CTL, cytotoxic T lymphocytes; LN, lymph node; MS, mass spectrometry; pMHCI, peptide bound to major histocompatibility complex class I molecule; TCR, T cell receptor.
